# The Emerging Adults Gambling Survey: study protocol

**DOI:** 10.12688/wellcomeopenres.15969.1

**Published:** 2020-05-26

**Authors:** Heather Wardle

**Affiliations:** 1 Faculty of Public Health and Policy, London School of Hygiene & Tropical Medicine, 15-17 Tavistock Place, London, WC1H9SH, UK

**Keywords:** gambling, gaming, emerging adults, protocol, longitudinal

## Abstract

The Emerging Adults Gambling Survey is a longitudinal survey of young adults aged 16-24 living in Great Britain. It aims to explore a range of gambling behaviours and harms among young adults and examine how this changes over time. It is part of a broader project funded by Wellcome into the gambling behaviours of young people and its relationship with technological change. Funding is currently available for two waves of data collection: the first collected in June/August 2019 (n=3549) and the second to be collected in June/August 2020. The second wave of data collection will also obtain information about the immediate impact of coronavirus on gambling behaviours. With a sample size of 3549 for Wave 1, this is one of the largest study of gambling behaviours among young adults to be conducted in Great Britain and is a resource for other researchers to draw on. Data will be deposited in the UK Data Archive upon completion of Wave 2 data collection and analysis.  This protocol is intended to support other researchers to use this resource by setting out the study design and methods.

## Introduction

Since the turn of the 21 ^st^ century, the provision of gambling opportunities has expanded rapidly. This is partly due to change in technological infrastructure, allowing the development of new methods of gambling as well as new gambling products (e.g., in-play betting markets reliant on the rapid communication of data to operate). This is also partly due to an ongoing process of liberalisation of gambling legislation, positioning gambling into mainstream leisure activities ( Light, 2007). This occurred in Great Britain with the 2005 Gambling Act (fully enacted in 2007) and is replicated in other jurisdictions, especially North America where online gambling is being legalised on a state-by-state basis. Most recently, the state of Michigan has permitted online sports betting and betting via mobile phones for the first time ( State of Michigan, 2019).

Young people are growing up in fundamentally altered environment for gambling, especially in Great Britain, where gambling, gaming and digital technology are increasingly intertwined ( Responsible Gambling Strategy Board, 2018). Those aged 16–24 are the first cohort whose adolescence and childhood occurred in an environment when online gambling was always freely available and gambling, generally, was heavily promoted and marketed. Some academics have argued that when regulatory or legislative changes occur, especially in expanding gambling opportunities, some populations respond by changing behaviour in the short term but adapt in the longer term by reverting to pre-change patterns of behaviour; the so-called “adaption” theory ( LaPlante & Shaffer, 2007). LaPlante and Shaffer noted that social context was vitally important when seeking to understand exposure effects. For those aged 16–24 living in Great Britain, this is likely to be even more pertinent: it is uncertain whether such adaptation occurs as this cohort have grown up knowing no other regulatory or legislative environment; they have no prior experience to revert to. This has led to increasing concern about the ‘normalisation’ of gambling among young people ( Responsible Gambling Strategy Board, 2018).

It is therefore vital to explore gambling behaviours among this cohort and how this may be changing over time. This Emerging Adults Gambling Survey focuses on those aged 16–24 as both the first cohort to fully experience the altered gambling landscape ushered in by the Gambling Act 2005 and because of their status as ‘emerging adults’. Coined by Jeffrey Arnett (2000), emerging adulthood describes an age group who he argues are both demographically distinct and distinct in terms of their subjective perceptions and identity exploration. Arnett (2000) identified a greater propensity for risk taking behaviour among emerging adults, arguing they may wish to obtain a wide range of experiences before settling into adult roles and responsibilities or that freed from parental constraints or adult responsibilities, they could engage in sensation-seeking experimentation (though notably, the 21 ^st^ century has seen a marked decline in some risk-taking behaviours among this cohort; IAS, 2016). Nonetheless, this age group currently represents a unique cohort experiencing a range of life transitions and changes, within a vastly altered gambling environment, and are thus worthy of greater attention.

A critical consideration is the experience of gambling harms. Gambling harms are the adverse impacts from gambling on the health and wellbeing of individuals, families and communities ( Wardle *et al.*, 2019). As noted by Wardle *et al.*, these harms are diverse, affecting health, wealth and relationships and may reflect an interplay between individual, family and community processes, exacerbating existing inequalities for some. Gambling harms are the consequences associated with gambling and thus extend more broadly than previously acknowledged ( Wardle *et al.*, 2019). Among young people, the harms from gambling have a further dimension affecting future opportunities and development either through the impact of their own gambling or the gambling of others ( Blake *et al.*, 2019). Academic insight into broader harms associated with gambling among young adults is sparse as is insight on the relationship between gambling and broader health and wellbeing, including suicidality.

In Britain, as elsewhere, monitoring of gambling behaviours has tended to focus on the measurement of gambling participation and problem gambling in repeat cross-sectional surveys, typically demonstrating stable patterns of behaviour at the population level ( Conolly *et al.*, 2018). These general population studies produce national statistics for all adults in Britain but do not have sufficient power to look at the youngest cohort individually. Furthermore, it is increasingly recognised that gambling behaviour that can be fluid and variable, where changes in intensity of engagement over time are the norm and patterns of problematic gambling unstable within the individual ( Breen & Hing, 2014; LaPlante *et al.*, 2008; Reith & Dobbie, 2013). Quantitative assessment of gambling behaviour change has highlighted how patterns of engagement can be episodic and transient, changing based on various social contexts and life events ( Slutske *et al.*, 2003; Slutske *et al.*, 2009). Slutske, Jackson and Sher’s *et al.* (2009) assessment of gambling trajectories among youth revealed a wide array of changes in behaviour despite overall prevalence rates staying constant. This tendency was observed among a follow-up study of people who held a loyalty card for British Bookmaker’s, whereby stable problem gambling prevalence rates for each wave of data collection masked a great deal of movement in and out of problem behaviour ( Wardle *et al.*, 2017). However, as Reith & Dobbie (2013) have also shown, individual level gambling trajectories over time are varied and whilst stasis may not be the norm, there are groups of people for whom gambling behaviours are habitual, embedded and constant. This diversity of experience has received scant attention, especially among policy makers and politicians who tend to focus on overall prevalence rates and not the fluctuating patterns in behaviour that underlie them. As now recognised by the UK Gambling Commission in its National Strategy to Reduce Gambling Harms, there is a need for longitudinal insight into the changing gambling behaviours of young adults ( Gambling Commission, 2019a). The Emerging Adults Gambling Study was design to meet this need.

Specific objectives are to investigate changes in the relationship between youth gambling behaviour, including harmful gambling outcomes, with use and integration of varying forms of digital technology, with specific emphasis on understanding the determinants of gambling behaviour, inequalities in behaviours and the factors associated with problematic gambling among younger people.

## Protocol

### Sample frame and size

In Wave 1, the Emerging Adults Gambling Survey collected data from 3549 participants aged 16–24 living in the UK. Participants were drawn from YouGov’s online panel of over one million people ( Kennedy *et al.*, 2016; Twyman, 2008). This has up to date information on the profile of each member, allowing subsets of panel members to be invited to participate according to certain characteristics. For this study, participants were eligible if they were aged between 16 and 24, living in the UK and had not taken part in any other YouGov study on gambling in the past year. Wave 2 data collection will occur one year later from Wave 1, in July/August 2020. The anticipated number of respondents is c.2400, representing a likely attrition rate of 32.5%. The baseline rate of problem gambling observed in Wave 1 was 3.69%. Assuming a between wave correlation of 0.5, we will be able to detect changes in problem gambling rates between Wave 1 and Wave 2 of +/- 0.3 percentage points (at 80% power). This sample size will allow changes in gambling behaviours and problem gambling to be detected for sub-groups of participants.

YouGov is a non-probability panel, with attendant issues of generalisability. However, when researching young people it has advantages in terms of sample coverage over probability methods: surveys using the Postcode Address File as a sample frame exclude many young people living in student halls of residence; surveys of students using sampling frames from university registers exclude those not in education and results may not be generalisable to non-student populations. The YouGov panel includes both, and the Emerging Adults Gambling Survey provides a closer estimate to national statistics of the proportion of young people not in education, employment or training than ‘gold-standard’ probability surveys like the Health Survey for England (see Table 1). Furthermore, the primary aim of this study is to explore behaviour change over time through multi-variate and between group analyses. Whilst the sample frame may have limitations in terms of generalisability, online panels have been recommended for use in studies with these aims ( Callegaro *et al.*, 2014).

**Table 1.** Profile of respondents to the Health Survey for England, 2017, Emerging Adults Gambling Survey and National Statistics data.

	Data source		
	Health Survey for England 2017 (unweighted)	Emerging Adult’s Gambling Survey (unweighted)	National Statistics data
**Sex** *			
Male	45.6%	45.8%	51.3%
Female	54.4%	54.2%	48.7%
**Ethnic group (18–24 only)** **			
White/White British	83.6%	86.8%	81.5%
Mixed	2.5%	3.6%	3.2%
South Asian	9.2%	7.2%	10.3%
Black	3.9%	1.5%	3.7%
Other	0.8%	0.9%	1.3%
**Region (England only)** *			
North East	7.6%	4.3%	5.0%
North West	15.6%	12.1%	13.3%
Yorkshire and the Humber	10.4%	10.8%	10.5%
East Midlands	11.7%	8.6%	9.0%
West Midlands	10.4%	9.7%	11.1%
East of England	13.1%	8.9%	10.1%
London	12.8%	17.1%	15.3%
South East	12.1%	16.4%	15.9%
South West	6.3%	12.1%	9.7%
**Economic activity** *** ^			
Not in Education, Employment or Training	3.4%	12.4%	11.5%

*National statistics computed from the Office for National Statistics 2018 Mid-Population Estimates. Available at https://www.ons.gov.uk/peoplepopulationandcommunity/populationandmigration/populationestimates/datasets/populationestimatesforukenglandandwalesscotlandandnorthernireland

**National Statistics estimates computed from 2011 Census, available at https://www.ethnicity-facts-figures.service.gov.uk/uk-population-by-ethnicity/demographics/age-groups/latest#age-profile-by-ethnicity

***National Statistics estimates from Office for National Statistics bulletin: Young People not in education, employment or training, 2019. Available at: https://www.ons.gov.uk/employmentandlabourmarket/peoplenotinwork/unemployment/bulletins/youngpeoplenotineducationemploymentortrainingneet/august2019

^ Because of differences in how the data were captured, it is not possible to provide equivalent estimates of the proportion of young people in each survey who are students, although it is likely that the YouGov panel over-represents student populations, as 39% of those over 18 were categorised as a student.

### Data collection procedures

For Wave 1, email invites to participate were sent by YouGov to a random selection of their panel members, stratified by region. This email asked them to take part in a survey, without advertising its content, and asked participants to click through to the bespoke study. The first page of the bespoke survey then described the aims and objectives of the survey and obtained consent. 93% of people who accessed this page went on to complete the survey. Wave 1 data were collected between June and August 2019. Wave 2 data will be collected in July/August 2020 following similar procedures.

The survey asked about gambling, gaming, social media use and health-related behaviours. The theoretical framework guiding the Wave 1 questionnaire development was the socio-ecological model adapted for gambling by Wardle *et al.* (2019). The Wave 1 questionnaire reflected the multiple levels which may influence behaviour, ranging from individual characteristics (such as impulsivity), to family and community influences (such as parental gambling behaviours, gambling of peers, area-level deprivation) and societal influences (for example, exposure to marketing and advertising). The Wave 1 questionnaire is provided as extended data ( Wardle, 2020).

The Wave 1 questionnaire was developed by Dr Wardle, reviewed by an expert panel of academics and refinements were made. A field pilot collected data from 62 participants in May 2019. Pilot responses were reviewed by the lead author and members of the YouGov team and changes agreed. The first 250 responses for the main data collection were further reviewed for consistency, accuracy of routing, and to establish timing thresholds for seriousness checks. Participants who completed the survey in less than one standard deviation of the mean completion time were removed: 39 participants were excluded from the final dataset for this reason. In Wave 1, missing data were minimal and excluded from analyses (except where explicitly stated).

Wave 2 data collection will follow similar procedures, using the Wave 1 questionnaire as its base and updating with further questions about behaviour change. In particular, it will add a suite of questions about changed circumstances and behaviours in the context of COVID-19 and the experience of lockdown conditions. The March 2020 classification of COVID-19 as a pandemic, precipitated unprecedented restrictions on people’s movements and interactions in public and private settings. With the closure of commercial and social venues and cancellation of major sports events, COVID-19 altered the gambling landscape worldwide. Industry responses have been to heavily promote unaffected products (online slots/casino games, esports, virtual events, all associated with high rates of problem gambling) and/or to incentivise people to start gambling. ‘Lockdown’ conditions also potentially heighten risk factors for gambling and gambling harms (through boredom, stress, anxiety, financial problems, loneliness). The Wave 2 questionnaire will aim to investigate the immediate impact of these changes upon the gambling behaviours of Emerging Adults.

### Measures

***Primary outcome measures***. Participants were asked to report whether they have ever gambled on a range of 18 different gambling activities legally available in Great Britain. They were also asked to report how old they were the first time they gambled on each and how often they had gambled on each in the past 12 months. This included questions about esports betting and in-play betting. Problem gambling was measured using the Problem Gambling Severity Index (PGSI), a validated tool for the identification of gambling problems ( Ferris & Wynne, 2001). This was administered to anyone who had gambled in the past 12 months and thus produces estimates of current gambling problems. The PGSI score ranges from 0–27; a score of 0 indicated non-problem gambling or non-gambling; 1–2 is low risk gambling; 3–7 is moderate risk gambling, and a score of 8 or more is indicative of problem gambling.

Personal wellbeing was captured using the harmonised Office for National Statistics (ONS) four-item measure of personal wellbeing ( ONS, 2016). This asks participants to rate on a scale of 0 to 10 their current levels of life satisfaction; whether they do things that they feel are worthwhile; how happy they felt yesterday, and how anxious they felt yesterday.

Questions about suicidality were adapted from the Adult Psychiatric Morbidity Survey (APMS) series ( McManus *et al.*, 2019). Participants were asked: ‘In the last 12 months, have you ever thought of taking your life, even if you would not actually do it?’ and ‘In the last 12 months, have you ever made an attempt to take your life, by taking an overdose of tablets or in some other way?’

***Other measures***. Questions about social media use and gaming were adapted from the Millennium Cohort Study, Wave 7 ( IOE, 2020), which asked people to report how often they engaged in both on a typical weekday, with response options ranging from less than half an hour to 7 or more hours a day.

A suite of questions were adapted from the UK Gambling Commission’s Youth Gambling Survey to ask about use of gambling-like mechanics within video games ( Gambling Commission, 2019b). This included whether the participant had paid money to open loot boxes; opened loot boxes with in-game currency; had bet skins on external websites or had bet skins within the game.

Further questions about gambling harms were adapted from the Ipsos Mori’s development work on measuring gambling harms among young people ( Blake *et al.*, 2019). This included questions relating to gambling’s impact on health, wellbeing and relationships. The 8-item Attitudes Towards Gambling Questionnaire was included to measure gambling attitudes and norms ( Canale *et al.*, 2016; Wardle *et al.*, 2011).

Social network influences were captured through a range of questions asking about whether parents, family members or friends gambled and, if so, how often.

Questions about exposure and influence of gambling advertising and marketing were adapted from the Young People’s Survey of Gambling Promotion ( MacGregor *et al.*, 2020). This included questions about awareness of advertising/marketing; experience of direct marketing; use of social media to follow gambling companies; and self-reported influence of marketing/advertising on gambling behaviours.

Impulsivity was measured using a shortened form of the Eysenck Impulsivity Scale which is validated for use among adolescents [ Eysenck & Eysenck, 1977; Wills *et al.*, 1998]. Participants were asked to respond on a five-point scale how true seven different statements about impulsivity are for them. Consistent with the approach taken by other studies, impulsivity scores are computed as the average of the seven questions ( Auger *et al.*, 2010).

Risky alcohol consumption was identified using the Modified Single Alcohol Screening Questionnaire (M SASQ) ( Canagasaby & Vinson, 2005). This consists of one item from the Alcohol Use Disorders Identification Test about frequency of consuming eight or more units of alcohol for men or six more units of alcohol for women in a single event in the past year. A score of three or more identifies higher-risk drinkers.

Perceived loneliness was assessed by one item from the Social Functioning Questionnaire ( Tyrer *et al.*, 2005). Participants were asked to assess with a four-category response (very much, sometimes, not often, and not at all) the extent to which they had felt “lonely and isolated from other people” in the previous two weeks.

Ethnicity was self-reported using the harmonised ONS ethnicity question. Age was captured in single age years. Local area level deprivation was measured using the respective English, Scottish and Welsh Indices of Multiple Deprivation (IMD) scores matched at the ‘Output Area’ and quintiled for analysis. Respondents were asked to report both their parents’ level of academic attainment. This was grouped by whether at least one parent had a degree or higher or whether both parents’ qualifications were lower than degree level. Housing tenure, marital status and employment status were also collected in accordance with harmonised ONS questions. Respondents were asked to report their household’s total income and their own personal income.

### Data analysis

A range of analyses are planned utilising both Wave 1 to explore within group behaviours and Waves 1 and 2 to look at behaviour change. Analyses planned using Wave 1 data include assessment of the relationship between problem gambling and suicide attempts. This will follow the analytical procedure used by Wardle *et al.* (2020) to replicate analyses among Emerging Adults. Analyses will also include investigation of the profile and relationship to problem gambling among those who engage in gambling-like mechanics within video games and problem gambling. Using hierarchical regression, this will explore the extent to which the relationship between gambling-like mechanics is accounted for by other potentially confounding variables, like broader gambling engagement or underlying personality traits such as impulsivity. Wherever possible, analyses will be conducted separately for men and women.

Behaviour change will be examined between Waves 1 and 2 by identifying those whose gambling (and problem gambling) behaviour remained the same (including those who were abstinent); those whose behaviours increased and those whose behaviours decreased between waves. Multi-variate analyses will examine the factors associated with each, with the referent group for each model being those whose behaviours were the same at Wave 1 and did not deviate. This replicates the analytical procedures used by Graham *et al.* (2019) when examining changes in smoking, alcohol consumption, fruit and vegetable consumption and physical activity. This will include controls for a standard set of socio-demographic and economic factors, like age and deprivation. From this the incidence of problem gambling among this group will be calculated.

All multi-variate analyses will be subject to diagnostic tests for multi-collinearity by calculating the variance inflation factors (VIF) of all independent variables. A VIF value of less than 2 will be used as the threshold to indicate the presence of collinearity or not. Analyses will be performed using the complex survey function in SPSS v21 and Stata v15 to adjust for weighted stratified survey design. These complex survey modules produce a Walds F-test as the default test of significance ( Rao & Scott, 1984). For bivariate analyses, this assesses the extent to which the independent variable (suicide attempts, for example) varies by the dependent variables (age or problem gambling status, for example) and is the test on which all p-values are based. All estimates will be weighted to match the age, sex and regional profile of 16 to 24 year olds living in Great Britain. Additional weights for non-response between Waves 1 and 2 will be computed. Analyses will use weighted data and controlled for the stratified survey design and true (unweighted) bases will be presented.

### Ethics

Ethical approval for the study was granted by London School of Hygiene and Tropical Medicine’s Ethics Review Panel (ref: 15960). All participants, regardless of study responses, were provided with a list of help and supporting services at the end of the survey should they wish to speak with anyone about the issues raised. Following the procedure used in the Adult Psychiatric Morbidity Survey, all participants reporting suicide attempts were directed to speak with their General Practitioner.

### Dissemination, engagement and data availability

Findings will be disseminated through a range of methods, including but not limited to: academic journal articles, conference papers, short briefing papers presenting lay summaries to key policy makers (including the Gambling Commission, Politicians on the All Party Parliamentary Group on Gambling Harms, the WHO gambling panel, of which Dr Wardle is a member), verbal presentation of results formally and informally to key stakeholders, engagement with national press and blogs/commentaries. Dr Wardle has a strong range of networks, through her role as Deputy Chair of the Advisory Board for Safer Gambling, which provides advice to government on gambling policy, and will leverage these networks to ensure impact.

Fully anonymised datasets, after disclosure checks, will be made available as a resource to others at the end of the grant award and will be deposited with the UK Data Archive. Until that point, data will be available on request from the author to allow adherence with Open Science principles.

***Study status***. At the date of writing (May 2020), data from Wave 1 had been collected and analysed and questionnaire content for Wave 2 data collection planned, with fieldwork to be completed in July/August 2020. Funding options to extend the study to include a third wave of data collection, in July/August 2021 are being explored. The project will be completed by the end of the fellowship in March 2022.

## Conclusions

The landscape for gambling has changed rapidly in the last decade, with attendant questions of impact on young people. Despite stated need for longitudinal studies to explore behaviour change among young adults, few studies worldwide have examined this. This study aims to help fill this gap by exploring changing gambling behaviours among Emerging Adults in Britain, who are the first cohort of British youth to have grown up within the vastly altered regulatory environment created by the implementation of the Gambling Act 2005. In addition, the gambling landscape has recently experienced a short-term shock through the impact of COVID-19 on business and sporting events. This study will enable some examination of the immediate impact of COVID-19 on gambling behaviours, though longer term assessment would also be desirable. It is anticipated that findings will be incorporated into policy and regulatory change, especially as the Conservative Government has committed to review the Gambling Act 2005 with focus on the increasing recognition of gambling as a public health issue. Understanding the impact of gambling on young people is a vital component of this.

## Data availability

### Underlying data

No data are associated with this article.

### Extended data

Open Science Framework: Emerging Adults Gambling Survey. https://doi.org/10.17605/OSF.IO/ND8WT ( Wardle, 2020)

This project contains the following extended data:

- Emerging Adults Wave 1 questionnaire.docx (Wave 1 questionnaire)

Data are available under the terms of the Creative Commons Attribution 4.0 International license (CC-BY 4.0).
