# Supplementary material for: The Emerging Adults Gambling Survey: study protocol
Source: Wellcome Open Res. 2020 May 26;5:102. [Version 1] doi: 10.12688/wellcomeopenres.15969.1 (PMC7312251; doi:10.12688/wellcomeopenres.15969.1)
Supplement: Peer review history

# Peer review: Reviewer response for version 1

**DOI:** 10.21956/wellcomeopenres.17513.r38835

**Published:** 2020-06-23

**Authors:** Max Abbott

**Affiliations:** 1 Gambling and Addictions Research Centre, Faculty of Health and Environmental Sciences, Auckland University of Technology, Auckland, New Zealand

This paper provides an overview of a two-phase prospective survey of young adults resident in Great Britain. The primary focus is on gambling behaviours and changes in gambling behaviours, and relationships between these measures and a range of health and other factors. The baseline data collection phase has been completed.

 The rationale for the study is well articulated and sound. The objectives are justified in terms of relevant background literature and developments in gambling availability and policy. Perhaps more specific hypotheses could have been stated a priori. If available, the introduction could be strengthened by inclusion of systemic and perhaps other recent reviews of youth gambling, especially reviews of prospective community surveys. Mention could have been made of the increasing convergence of gambling and gaming and its potential consequences for young people.  In paragraph three mention is made of the finding of recent marked declines in some risk-taking behaviours among adolescents and young adults. Reference should also be made to general population studies that have found marked declines in gambling participation in this age group. Of further relevance is the finding that while participation has dropped markedly, problem and at-risk gambling has plateaued. Indeed in two studies, in Sweden and Australia, significant increases in problem/at-risk gambling accompanied substantial participation reductions. This pattern of low participation and high harm has been found in a number of other population groups that are recently exposed to more 'toxic' gambling forms and/or are highly vulnerable to gambling-related harm for other reasons. These findings raise important questions for the present study.  

 The study design is appropriate and the methodology is generally well described, while appropriate, as indicated by author, the sample is non-representative of the population of interest. While weighting may assist extreme caution will be required in generalising study findings beyond the sample investigated. Related to this, detail is lacking with respect to the number of panel members who were randomly selected and the proportion that accessed the survey page. While a surprisingly high 93% of people who accessed the page went on to complete survey, what proportion of invitees accessed the page and what is the overall response rate? How representative are participants of the panellists selected and of the Great Britain youth population? The impact of COVID-19 will probable yield interesting and relevant information, while complicating interpretation of the findings. The analytic procedures are specified and appear to be appropriate for the study objectives and design.  On this matter I would, however, probably defer to a biostatistician with extensive expertise in the analysis of prospective data. I am confident that dissemination of the findings will be well addressed and note that anonymised datasets will be made available for other investigators to access.

Is the study design appropriate for the research question?

Yes

Is the rationale for, and objectives of, the study clearly described?

Yes

Are sufficient details of the methods provided to allow replication by others?

Yes

Are the datasets clearly presented in a useable and accessible format?

Not applicable

Reviewer Expertise:

I have decades of experience leading and contributing to general population and other studies of gambling and problem gambling/gambling harm. This includes leading large general population prospective gambling studies in a number of jurisdictions as well as clinical trials and mixed methods studies examining particular sociodemographic groups including young people, indigenous and migrant groups.

I confirm that I have read this submission and believe that I have an appropriate level of expertise to confirm that it is of an acceptable scientific standard.

# Peer review: Reviewer response for version 1

**DOI:** 10.21956/wellcomeopenres.17513.r38832

**Published:** 2020-06-16

**Authors:** Rachel Volberg

**Affiliations:** 1 Department of Biostatistics and Epidemiology, University of Massachusetts, Amherst, MA, USA

The article describes the study protocol for a proposed second wave of data collection from a large cohort of young British adults (aged 16-24 at Wave 1) regarding a range of gambling behaviors and related harms. The aim is to understand changes in gambling behaviors and harms over time among young adults and, additionally, obtain information about the impact of COVID-19 on the cohort members’ gambling behaviors.

 There is a clear rationale presented for the study. Young people in Britain are growing up in an environment where online gambling has always been freely available and heavily promoted. With no other experiential environment, it is unclear whether young people’s gambling behaviors will reduce over time as has occurred among older adults whose gambling experience encompasses more tightly regulated environments. Furthermore, monitoring of gambling behaviors over time has typically been accomplished through repeat cross-sectional surveys which tend to identify stable patterns of behavior at the population level. These stable patterns mask a great deal of underlying liability in gambling behaviors and experiences of gambling harms. The specific objective of the study is to investigate the relationship between youth gambling behavior and use of various digital technologies with a focus on the determinants of gambling behavior, inequalities in such behavior, and factors associated with problem gambling among young Britons.

 The study design appears appropriate and responsive to the specific objective of the study. The author specifies that Wave 1 was drawn from YouGov, which is a non-probability online panel with some issues of generalizability. The author anticipates a 32.5% attrition rate for final sample of ~2400. The Problem Gambling Severity Index was used to assess problem gambling at Wave 1 and is proposed again for Wave 2. While conventional scoring was used in Wave 1, it would be desirable to assess the impact of using a cutoff of 5+ on the PGSI since this aligns better with clinical assessments of problem gambling. The other measures described seem appropriate and all are chosen from existing validated measures.

 The methods are described in some detail. It is unclear from the article whether any incentives were offered to the respondents in Wave 1 or are proposed in Wave 2 to potentially reduce attrition. With regard to data analysis, it would be helpful to look at gambling intensity (time and money spent gambling) as well as gambling involvement (number of types of gambling engaged with) when considering potentially confounding variables.

 The data from both waves of the study will be deposited with the UK Data Archive at the end of the grant period. The author indicates that the data can be provided to other researchers upon request prior to the end of the grant period. There are no data associated with the article since this is a study protocol.

Is the study design appropriate for the research question?

Yes

Is the rationale for, and objectives of, the study clearly described?

Yes

Are sufficient details of the methods provided to allow replication by others?

Yes

Are the datasets clearly presented in a useable and accessible format?

Not applicable

Reviewer Expertise:

Gambling epidemiology and etiology; social and economic impacts of gambling; problem gambling measurement

I confirm that I have read this submission and believe that I have an appropriate level of expertise to confirm that it is of an acceptable scientific standard.
